# A Time-of-Flight Range Sensor Using Four-Tap Lock-In Pixels with High near Infrared Sensitivity for LiDAR Applications

**DOI:** 10.3390/s20010116

**Published:** 2019-12-23

**Authors:** Sanggwon Lee, Keita Yasutomi, Masato Morita, Hodaka Kawanishi, Shoji Kawahito

**Affiliations:** 1 Graduate School of Science and Technology, Shizuoka University, Hamamatsu, Shizuoka 432-8011, Japan; sglee@idl.rie.shizuoka.ac.jp (S.L.); mmori@idl.rie.shizuoka.ac.jp (M.M.); hkawa@idl.rie.shizuoka.ac.jp (H.K.); 2 Research Institute of Electronics, Shizuoka University, Hamamatsu, Shizuoka 432-8011, Japan; kyasu@idl.rie.shizuoka.ac.jp

**Keywords:** CMOS image sensor, SOI detector, backside-illumination, time-of-flight, lock-in pixel

## Abstract

In this paper, a back-illuminated (BSI) time-of-flight (TOF) sensor using 0.2 µm silicon-on-insulator (SOI) complementary metal oxide semiconductor (CMOS) technology is developed for long-range laser imaging detection and ranging (LiDAR) application. A 200 µm-thick bulk silicon in the SOI substrate is fully depleted by applying high negative voltage at the backside for higher quantum efficiency (QE) in a near-infrared (NIR) region. The proposed SOI-based four-tap charge modulator achieves a high-speed charge modulation and high modulation contrast of 71% in a NIR region. In addition, in-pixel drain function is used for short-pulse TOF measurements. A distance measurement up to 27 m is carried out with +1.8~−3.0% linearity error and range resolution of 4.5 cm in outdoor conditions. The measured QE of 55% is attained at 940 nm which is suitable for outdoor use due to the reduced spectral components of solar radiation.

## 1. Introduction

Recently, much attentions have been paid to complementary metal oxide semiconductor (CMOS)-based time-of-flight (TOF) range image sensors to be used for a variety of applications such as AR/VR/MR, security systems, drones, robots and autonomous vehicles [1,2,3,4]. These near-future applications require more distance in its measurement range and higher tolerance and resolution under high ambient light operation for outdoor use. There are two types of TOF range image sensors: direct and indirect TOF range image sensors. For a long-range laser imaging detection and ranging (LiDAR) application, direct-type TOF range image sensors are believed to be suitable. A single photon avalanche diode (SPAD) is a key device for CMOS-based direct TOF imagers with sufficient spatial resolution. Indeed, recently several SPAD-based LiDARs with measurable distance of over 100 m have been reported [5,6,7]. However, SPAD-based direct TOF sensors need relatively complicated circuits with high-speed clocking for time-stamp measurements of photons and statistical processing for removing the influence of ambient light and wide dynamic range, and, as a result, SPAD-based direct TOF sensors have a difficulty of having high spatial resolution, or large pixel number. Another issue of current SPAD-based imagers is low photon detection efficiency (PDE) at near-infrared (NIR) region. For outdoor use of TOF range imagers, a NIR band centered at 940 nm is useful for relaxing the influence of direct sun light where the spectral components of solar radiation. However, SPAD-based LiDARs with enhanced NIR sensitivity has often the PDE of less than 10% at 940 nm band [8,9]. On the other hand, current indirect TOF sensors based on lock-in pixels are useful devices for 3D depth imaging of a few meters or up to several meters [10,11,12]. As a 1Mpixel indirect TOF image sensor has demonstrated [13], indirect types are good for having high spatial resolution. Although the indirect TOF image sensors often suffer from multipath reflections and retro-reflector or associated stray light signals, numerous methods to mitigate those effects have been reported [14,15,16], and the subject is still developing actively. Using very high continuous-wave (CW) modulation frequency for demodulators in the TOF pixel, the depth resolution has been greatly improved if the distance is a few meters. However, the issue of indirect TOF imagers is its applicable distance, tolerance to ambient light, depth resolution under strong ambient light. The operation of 3D range image sensors under such aggressive conditions is increasingly demanded for the above-mentioned applications to be realized.

To address the issues for next-generation applications of TOF imagers, this paper proposes a backside illuminated (BSI) silicon-on-insulator (SOI) based four-tap lock-in pixel indirect TOF imager using short-pulse modulation [17]. The proposed indirect TOF pixel has multi-tapped gates for signal outputs and a high-speed charge draining gate and uses short light pulse (small duty) for TOF measurements [18,19]. This technique allows us to use range-shifted TOF measurements that cover wide distances while having relatively high depth resolution, even under strong ambient light, by exploiting the feature of power-concentrated short light pulse, short-time gating for demodulation, and draining ambient light components during the off-state of signal light pulse. For high quantum efficiency (QE) and high-speed carrier response, a thick substrate of 200 µm is used as a BSI photodetector, and is fully depleted by applying high back bias voltage to achieve high-speed charge modulation as well as high QE in a NIR 940 nm band. The SOI active layer is used for a gate structure to modulate a channel potential that realizes charge modulation. Recently, various BSI technologies with a fully-depleted thick substrate [20,21,22,23,24,25] have been reported. The SOI-based pixel detector (SOIPIX) structure used in this paper is one of such technologies, but suitable for simultaneously integrating X-ray or near-infrared photon detectors and high-density CMOS circuits in a pixel as a 3D stacking manner [25,26]. A proof-of-concept TOF sensor chip based on the SOIPIX technology has been implemented and tested. A high QE and resulting high range resolution in long-range TOF measurements have been demonstrated.

The remainder of this paper is organized as follows. Section 2 describes the pixel structure and operation. Section 3 treats TOF measurement algorithms using range-shifted short pulses. The results of implementation of a TOF sensor chip are given in Section 4. Section 5 presents concluding remarks.

## 2. Lock-In Pixel Structure and Operation

### 2.1. Silicon-On Insulator (SOI) Lock-In Pixel Detector

**Figure 1:**
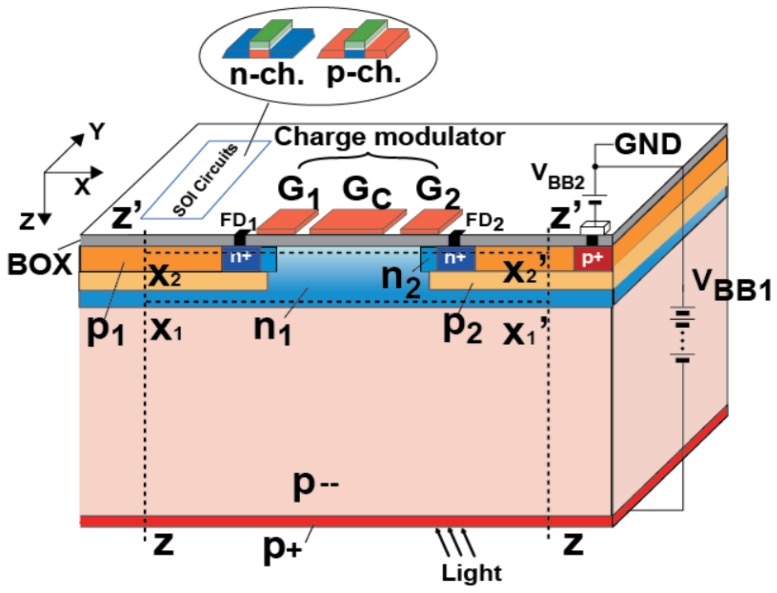
Proposed silicon-on-insulator (SOI)-based lock-in pixel detector structure.

The SOI-based lock-in pixel detector as shown in Figure 1 is based on the SOIPIX technology [25,26,27] and realized by adding a photo-charge modulation structure implemented by highly-doped thin Si (SOI) as the gate, the BOX (buried oxide) as the insulator, and the substrate Si as the semiconductor layer. Although the thin SOI layer is basically used for CMOS readout circuits, in the charge modulator area the Si active layer with n+ highly doped and typically used for a source or drain of SOI transistors forms a transfer gate. In the front surface region of the substrate in the charge modulator area, the buried n-well (n_1_) layer is formed for creating a buried channel of the charge modulator. The structure for the charge modulation is illustrated based on a two-tap lock-in pixel for simplicity. The SOI transfer gates (G_1_ and G_2_) modulate the channel potential for high-speed lock-in detection. A constant negative bias is applied to the center gate (G_C_) in order to make the front-surface pinning to the potential of the front-surface bias, *V_BB2_*. For the stable operation of SOI circuits, the rest of the area is occupied with the buried p-well layers (p_1_) under the BOX surface. In order to reduce dark current and carrier trapping at the Si-SiO_2_ interface and simultaneously collecting photo-carriers generated at deep inside of substrate Si, stacking of two buried p-wells (p_1_ and p_2_), and buried n-well (n_1_) is used at this region other than the area of the charge modulator. The p_1_ layer has a relatively high doping concentration that is around 10^5^–10^6^ times higher than the substrate (p--). It neutralizes the front surface of the substrate for pinning the potential to the front-surface bias (*V_BB2_*). This layer also acts as the shielding from the photo-charge detector of the substrate to SOI circuits. The p_2_ layer creates a potential barrier under the n+ floating diffusions (FDs) to reduce the parasitic photo-sensitivity of FDs. The n_1_ created under p_2_ is fully depleted and acts as a buried channel for horizontally drifting photo-carriers which are drifted to the channel from deep inside of the substrate by the vertical electric field when the reverse bias is applied at the back substrate contact (p+). This n_1_ layer is also used for preventing a punch-through hole current from the buried p-wells to the substrate by creating a potential barrier to holes in the p-wells.

**Figure 2:**
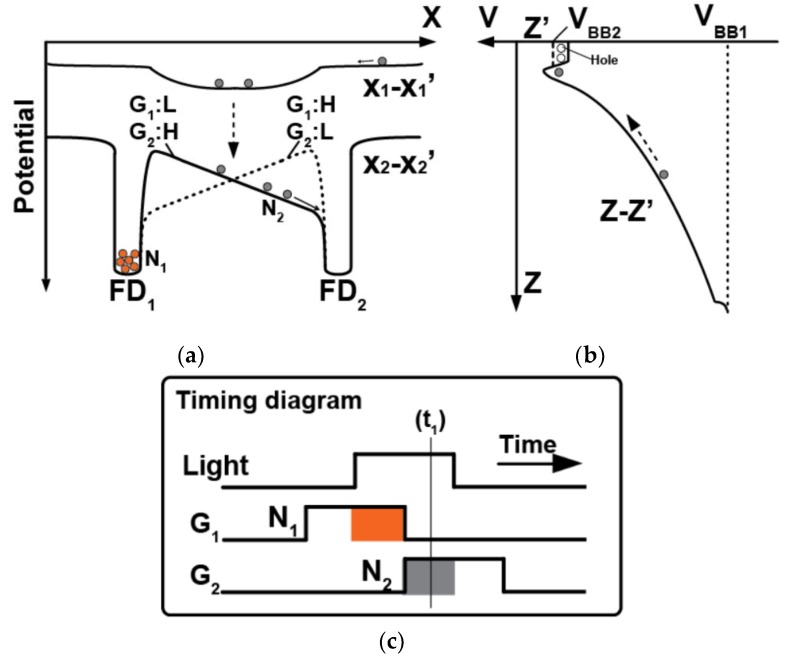
Concept of potential design and its timing diagram for the proposed SOI-based lock-in detector (two-tap). The potential distributions (**a**) for X_1_ − X_1_′ and X_2_ − X_2_′ axes (**b**) for Z − Z′ axis (See Figure 1). Those potential distributions are drawn at the timing of t_1_ in (**c**).

Figure 2 shows the conceptual potential diagram for the SOI-based lock-in pixel detector. By applying high negative backside bias (V_BB1_), a vertical electrical field is formed as shown in the right-side potential distribution on Z − Z′ (in Figure 2b). The photo-electrons generated at the deep inside of the substrate are accelerated toward the buried channel (n_1_). Because of the potential barrier by the p_2_/n_1_ junction and the lateral electric field created in the depleted buried channel (n_1_), the electrons are transferred to the central region of the pixel along the potential distribution on X_1_ − X_1_′ and then to the front surface just under the transfer gates. The photo-electrons generated at the deep inside of substrate just under the charge modulator are directly accelerated to the channel region of the charge modulator. Depending on the applied gate voltage to the transfer gates, G_1_ and G_2_, a potential slope to accelerate carriers to left to right or right to left is created at the buried channel as shown in the left-side potential distribution on X_2_ − X_2_′ (in Figure 2a). By applying the gating pulses for G_1_ and G_2_ consecutively as shown in the timing diagram of Figure 2c, the signal *N*_1_ (orange colored) as a result of overlapping of light pulse with the gating time of G_1_ is accumulated in the FD_1_ node and the signal *N*_2_ (gray colored) due to that of G_2_ is accumulated in the FD_2_ node. Since the difference between *N*_1_ and *N*_2_ depends on the delay of the light pulse, or the TOF, the TOF can be measured by knowing the charge amounts of *N*_1_ and *N*_2_. The details are described in Section 3 for the case of four-tap lock-in pixels.

### 2.2. SOI-Based Four-Tap Lock-In Pixel for TOF Sensors

**Figure 3:**
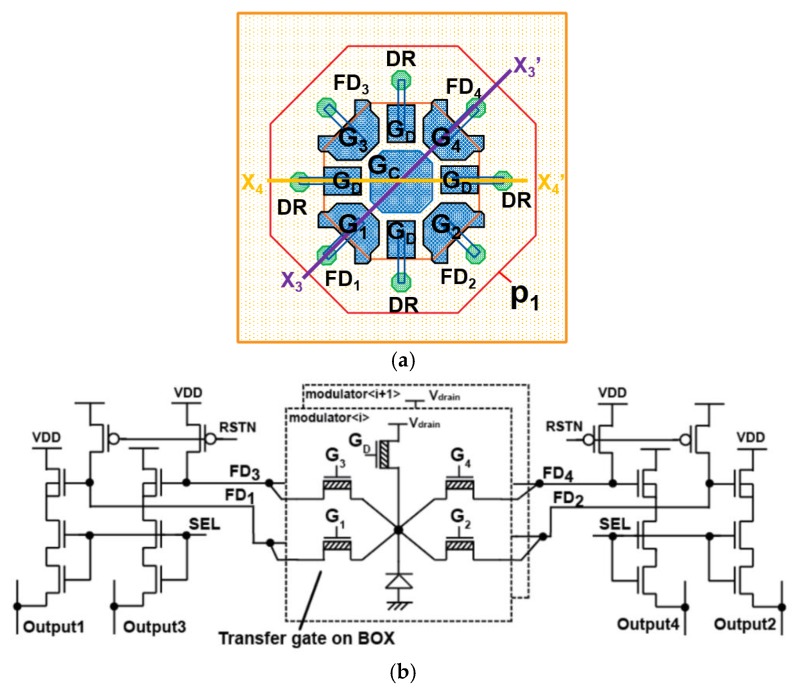
Developed four-tap lock-in pixel. (**a**) Pixel layout and (**b**) its equivalent circuit.

Figure 3a shows the top view of the SOI-based four-tap lock-in charge modulator. It has four transfer gates (G_1_, G_2_, G_3_, and G_4_) for transferring those time-gated signals and drain gates (G_D_) for draining unwanted photo-generated electrons. Generated and gathered signal charges at the front-surface of the charge modulator are transferred to one of four FDs, i.e., FD_1_, FD_2_, FD_3_, or FD_4_ by applying high voltage level to one of four transfer gates, G_1_, G_2_, G_3_, or G_4_, and low voltage level to the others. For draining signal or unwanted charges, G_D_ is set to high level and the other gates are set to low level.

As shown in Figure 3b, the unit pixel consists of two charge modulators whose FD nodes are connected in parallel, hence charge signals of the same node name are summed in the charge domain. The source of pMOS reset transistors and the gate of source follower transistors are connected to the four FDs, and together with select transistors, 4-channel 3T-type active pixel readout circuits are implemented for each pixel. The maximum rating voltage (VDS) of nMOS is 2.5 V in the SOI technology. To guarantee that the VDS of transistors is smaller than the maximum rating, the two nMOS transistors are connected in series for the select transistors. The size of charge modulator is 18 × 18 µm^2^ and the area of a unit pixel including two modulators is 36 × 18 µm^2^. The photo-charge generated in the substrate detector within the area of 36 µm × 18 µm is collected in either one of the two modulators and the modulated charges of them are summed up. In the SOI layer, the structure for the two modulator units occupies 54% of the area of 36 µm × 18 µm and the other area (46%) is used for readout circuits including reset, select and source follower transistors and interconnections. Therefore, without loosing the 100% fill-factor, the two modulator units and readout circuits are implemented in the area of 36 µm × 18 µm. The pixel size is chosen because the size of the readout circuit is designed with 9 µm.

**Figure 4:**
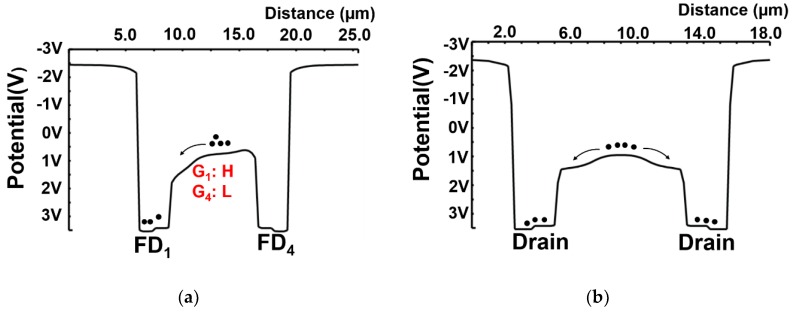

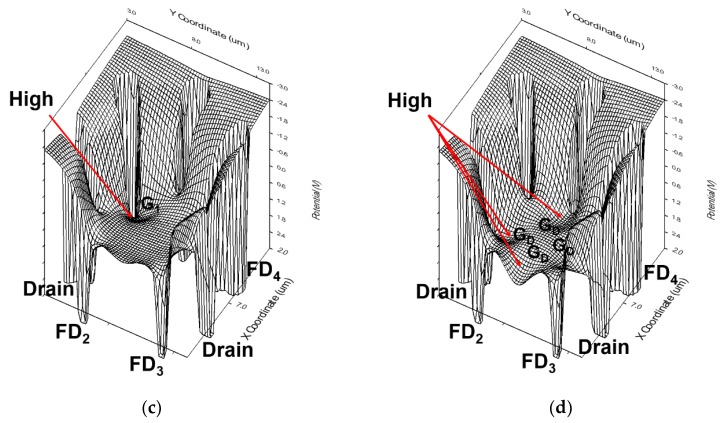
Simulation results for potential diagrams of a four-tap pixel. (**a**,**c**) G_1_ = high, G_2_, G_3_, G_4_; G_D_ = low (Transfer mode) (**b**,**d**) G_D_ = high; G_1_, G_2_, G_3_, G_4_ = low (Drain mode).

The simulated potential diagram of the designed four-tap lock-in pixel along cross-sections X_3_ − X_3_′ and X_4_ − X_4_′ in Figure 3a and 3D potential distributions are shown in Figure 4. Figure 4a,c show a charge transfer mode by G_1_ and Figure 4b,d show a charge drain mode by G_D_. For the transfer mode by G_1_, the gate voltage of G_1_ is set to high and those of the other gates (G_2_, G_3_, G_4_, and G_D_) are set to low. For the drain mode, G_D_ is set to high and the other gates are set to low. In these simulations, the thickness of the BOX layer is set to 200 nm and the V_BB2_ potential of −3.5 V is applied. Even with the thick BOX layer, potential modulation in the channel is observed. Since any potential barriers or dips are not observed, a high-speed charge modulation is expected.

**Figure 5:**
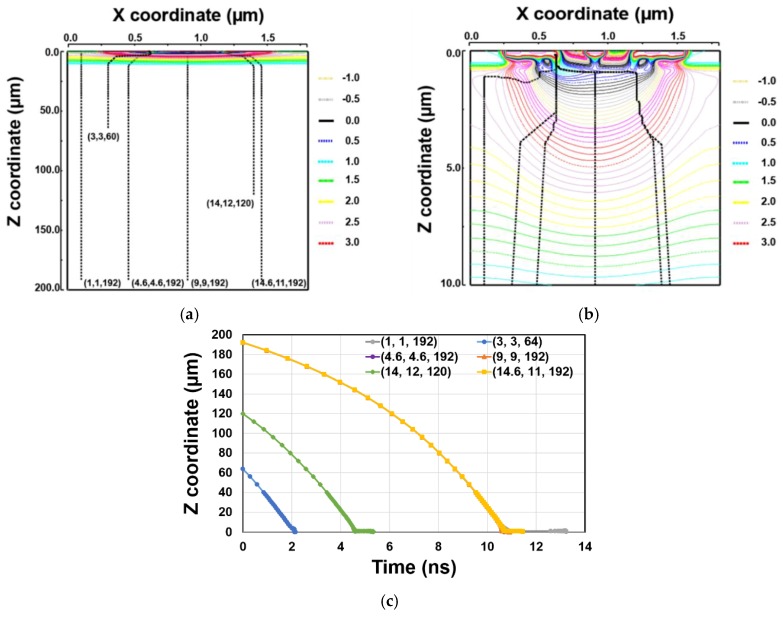
Simulation results for charge transfer. (**a**) Z = 0–200 µm. (**b**) Z = 0–10 µm (zoomed). (**c**) Charge transfer time to FD_1_ node. Electrons are placed at (1, 1, 192), (3, 3, 60), (4.6, 4.6, 192), (9, 9, 192), (14, 12, 120), and (14.6, 11, 192).

The electron trajectories of generated signal and their charge transfer time are shown in Figure 5. In the simulations, signal electrons are placed on different initial points: (X, Y, Z) = (1, 1, 192), (3, 3, 60), (4.6, 4.6, 192), (9, 9, 192), (14, 12, 120), and (14.6, 11, 192), where G_1_ is set to high. Figure 5a shows the electron trajectories from 0 to 200 µm and Figure 5b shows its enlarged figure from 0 to 10 µm. Figure 5c shows the charge transfer time from the initial positions to FD_1_. From the simulation result of Figure 5, the generated electron is transferred to the FD_1_ within 15 ns.

**Figure 6:**
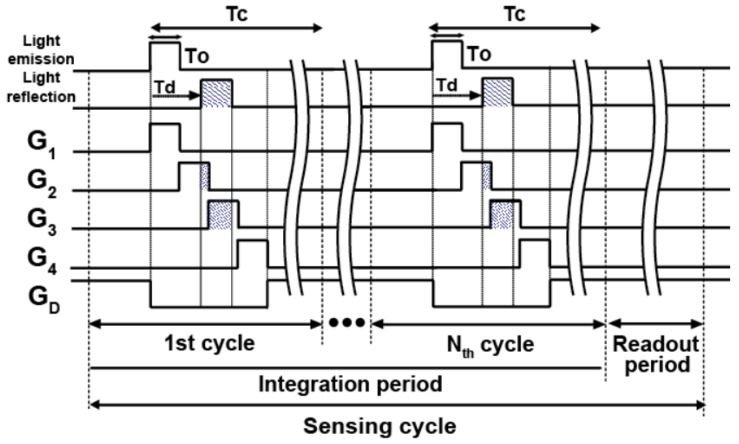
The timing diagram for the four-tap lock-in pixel.

Figure 6 shows the timing diagram for the four-tap lock-in pixel operation. One sensing cycle operation includes an integration period and the readout period. Each integration period contains *N* lighting cycles with the period of *T_C_* and in each lighting cycle, four transfer gates are opened consecutively with the width of *T*_0_ and the rest of time are used for photo-charge draining by opening the drain gate. To obtain a sufficiently large signal for calculating depth resolution, the number of lighting cycles, *N*, is increased. During the readout period, the G_D_ is always set to high and the other gates are set to low to drain unexpected signals. The accumulated signals transferred at FD nodes (FD_1_, FD_2_, FD_3_, and FD_4_) are read out through the in-pixel source followers. By using four output signals, the depth calculation is carried out. The details are described in Section 3.

## 3. TOF Range Calculation and Resolution with Four-Tap Lock-On-Pixel and Short Pulse Modulation

**Figure 7:**
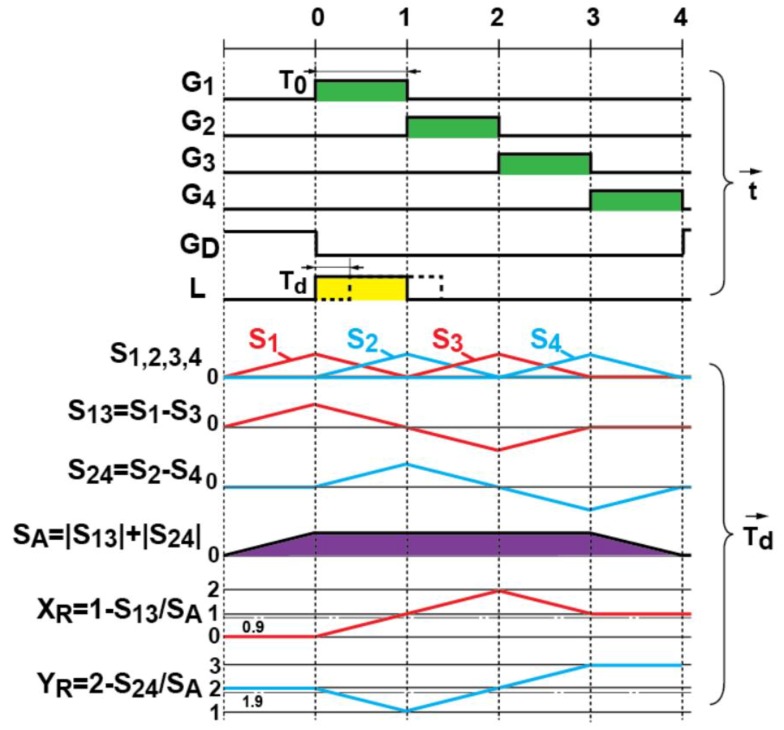
The timing diagram for the gating and the corresponding response of the signal outputs to the time-of-flight (TOF) of the light pulse, *T_d_*.

Figure 7 shows the timing diagram for the gating and the corresponding response of the signal outputs to the TOF of the light pulse, *T_d_*. The pixel output signals, *S*_1_, *S*_2_, *S*_3_, and *S*_4_, respond to the light pulse delay with a triangular shape as shown in Figure 7. Therefore, the difference of *S*_1_ and *S*_3_, *S*_13_ = (*S*_1_ − *S*_3_) and *S*_24_ = (*S*_2_ − *S*_4_) respond linearly to the TOF of light pulse within a range of 2*T*_0_. The functions of *T_d_*, calculated by the difference of the signals gated by G_1_ and that by G_3_, i.e., *S*_13_ and gated by G_2_ and that by G_4_, i.e., *S*_24_ normalized by the signal amplitude *S_A_* = (|*S*_13_| + |*S*_24_|) are expressed as:

(1) $$X_{R}=1-\frac{S_{13}}{S_{A}}$$

and

(2) $$Y_{R}=2-\frac{S_{24}}{S_{A}}$$

respectively. *X_R_* responds linearly to *T_d_* from 0 to 2*T*_0_ and *Y_R_* responds linearly to *T_d_* from *T*_0_ to *3T*_0_. By combining these two responses to *T_d_*, the depth range corresponds to the TOF range of 3 times *T*_0_ can be measured as:

(3) $$D=D_{TW}\left\{X_{R}(1-Z)+Y_{R}Z\right\}$$

where *D_TW_* = (0.5*cT*_0_; *c* is the velocity of light) is the unit depth determined by the light pulse width and *Z* is a factor of choosing *X_R_* or *Y_R_* or blending *X_R_* and *Y_R_* expressed as:

(4) $$Z=\left\{ \begin{array}{ll} 1 & \left(if\;Y_{R}\geq 1.9\&X_{R}\geq 0.9\right)\\ 0.5 & \left(if\;Y_{R}<1.9\&X_{R}\geq 0.9\right)\\ 0 & \left(if\;X_{R}<0.9\right) \end{array}\right.$$

The measurable range (*D_TW_*) is proportional to the pulse width of light source as shown in Equation (3), while the range resolution is inversely proportional to the pulse width as described later. As a result, the short pulse width leads to a better range resolution and reducing background light influence at the cost of a reduced measurable range. The pulse width can be adjusted arbitrarily depending on the required range resolution determined by its application.

## 4. Results and Discussion

### 4.1. Implemented TOF Sensor Chip

**Figure 8:**
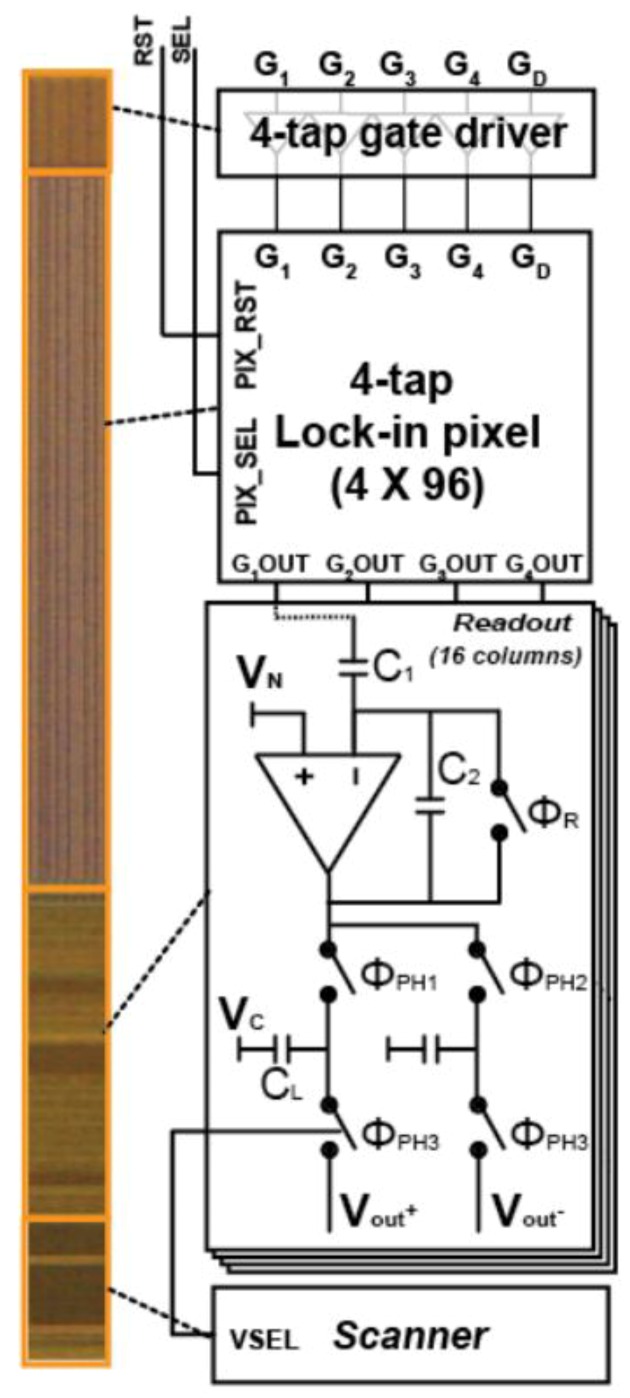
Chip micrograph and block diagram of the designed prototype sensor.

A prototype TOF range sensor with the proposed four-tap lock-in pixel is implemented using a 0.2 µm SOI detector technology [17]. The die micrograph and block diagram of the implemented chip are shown in Figure 8. Since the TOF sensor tested here is implemented in a part of a multi-purpose CIS chip, the chip area relevant to this paper only is shown in Figure 8. The TOF sensor is comprised of a four-tap gate driver, a small pixel array with 4 × 96 pixels, a readout circuit with 16 columns, and a scanner. Figure 8 also shows the schematic of the readout circuit. The readout circuit consists of a switched-capacitor amplifier (first stage) using two capacitors, C_1_ and C_2_, and two sample-and-hold circuits (second stage) using two capacitors, C_L_. Using the first stage, the pixel fixed pattern noise (FPN) is cancelled and the signal component is amplified by the capacitor ratio of C_1_/C_2_. In the second stage, the reset level and signal level of the first stage output are sampled in C_L_ and the FPN of the first stage is cancelled. The sampled reset and signal levels at each column are sequentially read out using a scanner by connecting them to final buffer amplifiers sequentially. The difference of the two outputs is taken and digitized by an external 14-bits analog-to-digital converter (ADC).

### 4.2. Measurement Results

**Figure 9:**
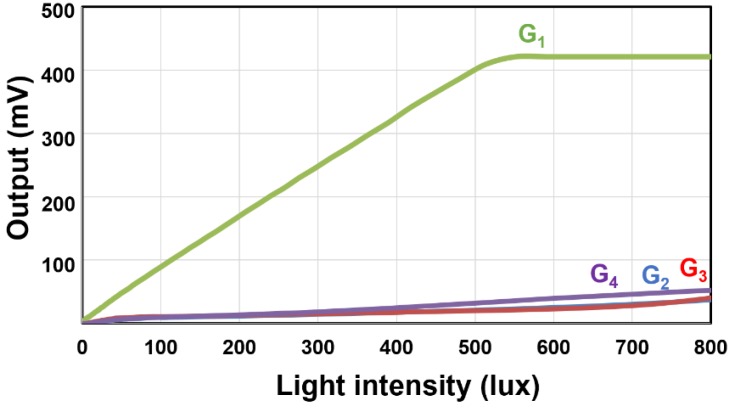
Photo-response as a function of light intensity. (at G_1_ = High and G_2_, G_3_, G_4_ = Low).

Figure 9 shows the measured photo-response of the four-tap lock-in pixel as a function of light intensity. A white light source box (Kyoritsu, LB-8623) is used. In this measurement, the G_1_ is always set to high and the other gates (G_2_, G_3_, and G_4_) are always set to low during the exposure period. A linear response to light intensity for the G_1_ before it is saturated is obtained. The parasitic sensitivity to the other signals is due to light or charge leakage. From this result, the extinction ratio defined by (*S*_1_/(*S*_1_ + *S*_2_ + *S*_3_ + *S*_4_)) × 100% measured at the signal level of 90% of the saturation is 84.7%.

**Figure 10:**
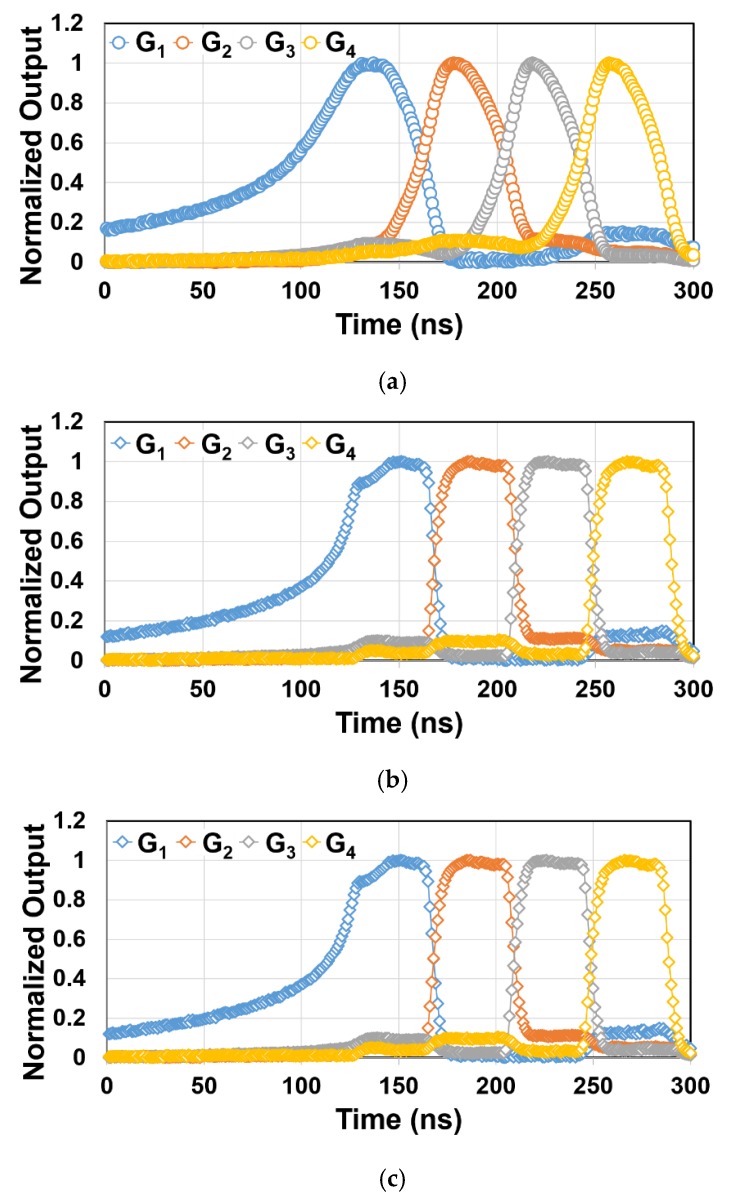
Modulation characteristic with various backside voltage using a 930 nm short pulse laser (**a**) V_BB1_ = −20 V, (**b**) V_BB1_ = −30 V, (**c**) V_BB1_ = −40 V.

Figure 10 shows the measurement results for modulation characteristics with different backside voltage (V_BB1_). In this measurement, a 930 nm short-pulse laser (LDB-160B manufactured by Tama Electric Inc.) is used as the light source and is irradiated to the pixel. The pulse width and the cycle time of the laser are set to <100 ps and 520 ns, respectively. The delay time of the laser trigger is scanned from 0 to 300 ns with the step of 1 ns. The gate pulse width of the pixel is set to 40 ns. For each measurement point on the delay, the number of irradiated light pulses is set to 50. The V_BB1_ is set to −20 V, −30 V, and −40 V. The ideal response is a rectangular shape to the pulse delay. For V_BB1_ of −20 V (Figure 10a), the response to the light pulse delay is much distorted because of the slow photo-carrier response, indicating the substrate is not fully depleted but partially neutralized. For V_BB1_ of −30 V (Figure 10b) and −40 V (Figure 10c), the response to the light pulse delay has a rectangular shape, indicating that the substrate is fully depleted by the backgate bias voltage of <−30 V, and photo-carriers are acquired speedily by drift of the carriers in the depletion region. The modulation contrast averaged for all gates, C_M_, is calculated by:

(5) $$C_{M}=\frac{1}{4}\sum_{i=1}^{4}\left[\max(\frac{{2S}_{i}-S_{\mathrm{SUM}}}{S_{\mathrm{SUM}}})\right]$$

where S_SUM_ is the sum of gates (S_1_ + S_2_ + S_3_ + S_4_). For the V_BB1_ of −30 V, the modulation contrast of 71% is obtained.

As shown in Figure 10, the signal due to the G_1_ gating for all the backgate bias voltages has a long preceding tail in the response to the delay. This indicates that the drain is not well functioning. The major reason for this is that the drain terminal in the chip is floating. As a result, in the draining phase, the unwanted signal charge is remained in the buried channel due to the lack of drain potential and then the charge is going to the FD_1_ when the G_1_ gate is opened. This problem can be solved by re-designing of the chip that the drain potential is fixed.

**Figure 11:**
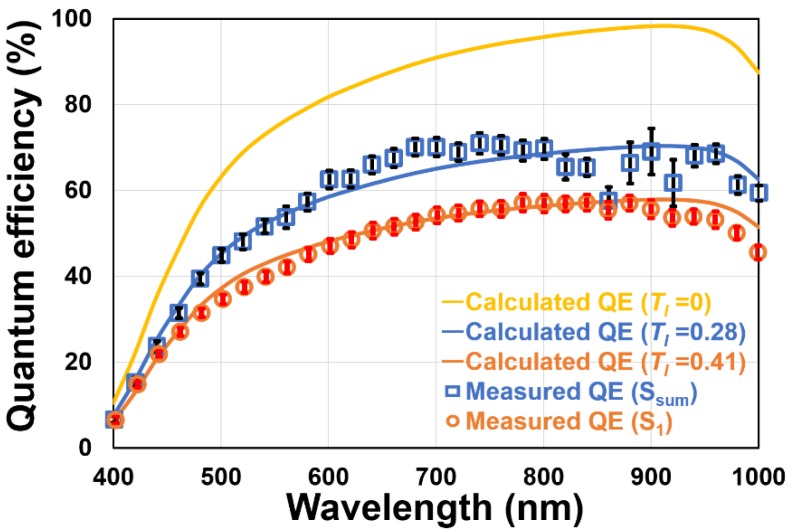
Measured quantum efficiency from the visible region to the near-infrared (NIR) region. The ideal quantum efficiencies (QEs) with various attenuation factors (T_l_) are calculated by Equation (6). S_SUM_ is the sum of signals from all the gates (S_1_ + S_2_ + S_3_ + S_4_).

Figure 11 shows the measured quantum efficiency (QE) from the visible to the NIR regions with error bars for every wavelength and the ideal QE curves with attenuation factors (T_l_). In this measurement, the G_1_ gate is always turned on and the other gates (G_2_, G_3_, G_4_, and G_D_) are turned off. The QE measured by the signal due to the G_1_ gating only has an influence of sensitivity loss due to the charge and light leakage to the other gates, or parasitic sensitivity of the other gates. This QE including the gating efficiency determines the TOF sensor performances such as depth resolution and is regarded as an effective QE. The QE measured with the sum of all the signals due to G_1_, G_2_, G_3_, and G_4_ gating is also shown in Figure 11 for a reference and the ideal QE curves are plotted. The ideal QE is calculated by:

(6) $$QE(\lambda )=\frac{\left(1-T_{l}\right)}{L_{a}(\lambda )}\int_{z_{1}}^{z_{2}}\exp(-\frac{z}{L_{a}(\lambda )})dz$$

where *L_a_*(*λ*) is an absorption coefficient of the silicon and *Z*_1_ and *Z*_2_ are minimum and maximum depths of the depletion layer from the backside of the substrate. *T_l_* is an overall attenuation factor including a loss due to a surface light reflection and a signal acquisition loss due to the imperfect charge modulation. In the fitting calculation, Z_1_ and Z_2_ are assumed to be 0.45 µm and 198 µm from the doping profile and the substrate thickness, and the least-square method is used where the data of overall wavelength are taken into account. The T_l_ of S_1_ and S_SUM_ are estimated to 0.41 and 0.28, respectively. In Figure 11, the QE calculated with no attenuation factor (T_l_ = 0) is also shown. Since the QE calculation is based on a simple assumption in which the attenuation factors are supposed to be independent of wavelengths, the fitting results are not always the same as the measurement results. However, the overall curve is similar to the ideal QE calculation. A high QE of 55% at 940 nm is obtained with S_1_ and a QE of >65% is obtained with S_SUM_. With an improvement of the design by reducing the parasitic sensitivity of the ungated outputs, a 10% higher effective QE will be realized.

### 4.3. Distance Measurement

**Figure 12:**
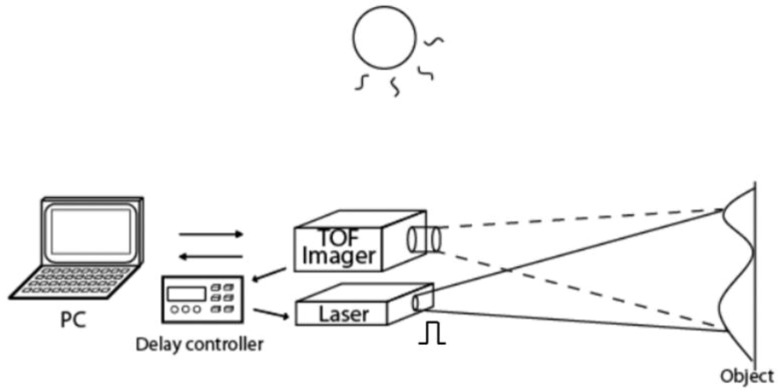
Setup of the implemented TOF sensor for depth measurement. The laser emission is controlled by the delay controller synchronized with the TOF imager.

**Figure 13:**
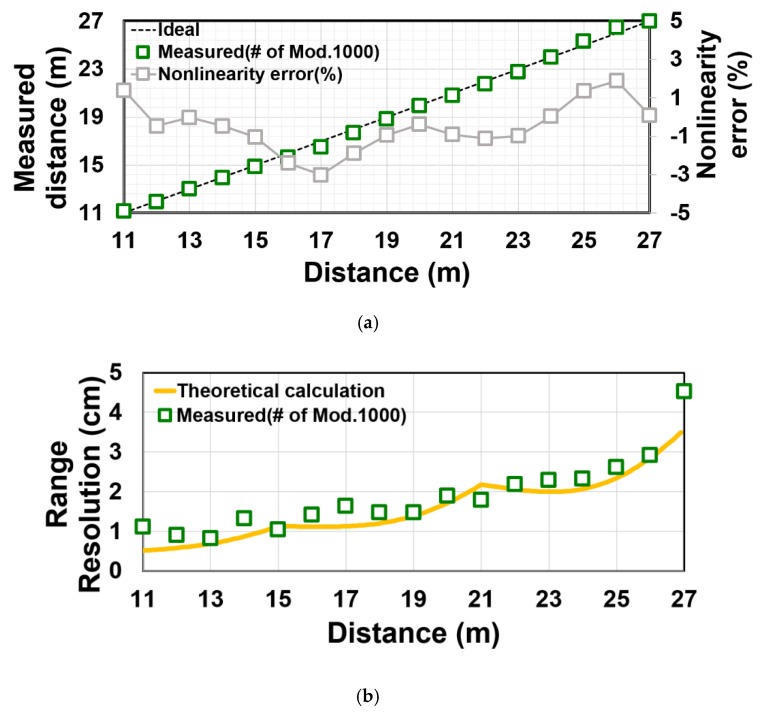
Distance plot: (**a**) Measured and ideal; (**b**) measured and calculated range resolution.

Figure 12 shows a measurement setup of the implemented TOF sensor for depth measurements. The laser pulse is synchronized with the TOF imager via a delay controller and is emitted to an object. Figure 13a,b show the measured distance and range resolution as a function of actual distance from 11 m to 27 m with a 1-m step. The measurement is done in outdoor and the background light level due to sun light is 75klux at the object plane. A 940 nm laser with an averaged light power density of 7.2 W/m^2^ (1.8 W peak) at 30 m and a target with high reflective white paper are used for the long-distance measurement. An optical bandpass filter at 940 nm with 10 nm full width at half maximum (FWHM) is used. In this measurement, the pulse width for both the light and gate pulses is set to 40 ns, which corresponds to a unit measurable range of 6 m in a one time-window. The number of lighting cycles (shown in Figure 6) is 1000 with the cycle time of 520 ns and the TOF measurement time is 520 µs. The setting of pulse width and the laser cycle is to use a small duty light pulse. The use of small duty cycle allows us to have a high signal-to-noise ratio under high ambient light and given average light power. The use of a large cycle time (small duty cycle) is also necessary for avoiding an ambiguity of TOF measurements. By using the four-tap lock-in pixel structure, three time-windows (totally 18 m) can be measured as described in Section 3. By using a gating-time offset, the measurable range can be shifted arbitrarily. This technique is called a range-shift method [18]. Using a gating-time offset of 6.7 ns, for example, the range from 1 m to 18 m can be measured. Using the gating-time offset of 60 ns, the range from 9 m to 27 m can be measured. In Figure 12, the gating-time offset of 60 ns is used and because of the limitation of our measurement setup, the measurement result for the range from 11 m to 27 m only is shown. In the present measurement setup, there is a difficulty of accurate measurements of distance below 11 m. This is because the implemented TOF sensor has a very small number of horizontal pixels (4 pixels), the spread angle of the laser used in the distance measurement is also relatively small. As a result, the alignment of the reflected light to the pixel areas becomes difficult, particularly for the closer distance.

In Figure 13a, the distance nonlinearity error is measured to be +1.8~−3.0%. Figure 13b shows the measured and theoretically calculated range resolution. The theoretical range resolution, $\sigma_{D}$, within one time window is given by

(7) $$\sigma_{D}=\frac{D_{TW}}{C_{D}\sqrt{N_{S}}}\times \sqrt{\frac{C_{D}N_{TOF}(N_{S}-N_{TOF})}{N_{S}^{2}}+2(1-2\frac{N_{TOF}}{N_{S}}+2\frac{N_{TOF}^{2}}{N_{S}^{2}})(\frac{1-C_{D}}{4}+\frac{N_{a}}{N_{S}}+\frac{\sigma_{R}^{2}}{N_{S}})}$$

where *N_S_* is total signal electrons, *N_TOF_* is signal electrons associated with TOF, *N_a_* is the background light signal, $\sigma_{R}$ is the dark noise. The derivation of Equation (7) is similar to [3,18]. Unlike in [3], a factor of modulation contrast, *C_D_*, is included in Equation (7). The *C_D_* takes a value from 0 to 1 and ideally equals 1. The number of total signal electrons (*N_S_*) is expressed as

(8) $$N_{S}=\frac{\lambda }{hc}\times QE\times A_{pix}\times FF\times T_{a}\times \frac{P}{W\times H}\times \frac{RT_{L}}{4F_{N}^{2}}$$

where *h* is the plank constant, *A_pix_* is the area of the pixel, *FF* is fill factor of the pixel, *T_a_* is the integration time, *P* is peak power of the laser source, *P*/(*W* × *H*) is power density on the sensor plane through a lens with transmission efficiency (*T_L_*) and F-number (*F_N_*) by an object with reflectivity (*R*).

**Figure 14:**
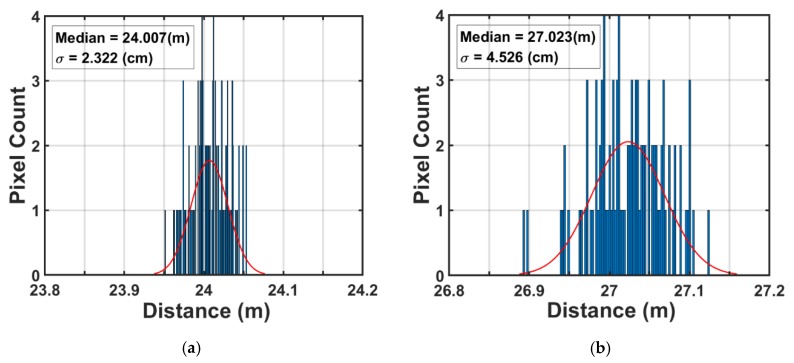
Histogram of distance at (**a**) 24 m and (**b**) 27 m.

The measured range resolution, which is also called the standard deviation of depth or depth noise, is 4.5 cm at 27 m. The measurement result of range resolution at 27 m suddenly worsen compared to the theoretical calculation. A possible reason for this is the jitter on the light pulse. The point of 27 m corresponds to the edge of measurable range, where the reflected light pulse is integrated by G_4_ gate only or a part of the reflected light pulse is drained by G_D_. Although the jitter of the light trigger is also induced at the transition edge of G_1_–G_2_, G_2_–G_3_, or G_3_–G_4_, the behavior and the resulting influence at the edge of measurable range is expected to be different from the others. Figure 14 shows the histogram of distance at (a) 24 m and (b) 27 m. Because of the jitter or other unexpected noise sources, the distribution is not a simple Gaussian distribution at 27 m. The sensor performance and characteristics are summarized in Table 1.

## 5. Conclusions

This paper presents a TOF range imager with SOI-based lock-in pixels for long-range outdoor applications. The proposed TOF range imager has fully depleted bulk silicon for the backside illumination to enhance the responsivity in the NIR region. By using the SOI technology, a 200 µm thick substrate is used as a photodetector and the SOI active layer is used for the transfer gate to modulate the channel potential. The implemented four-tap lock-in pixel structure demonstrates a high-speed charge modulation and high modulation contrast of 71% at 40 ns gate width. The prototype TOF chip also demonstrates the distance measurement up to 27 m with +1.8~−3.0% linearity error and the measured range resolution of less than 4.5 cm using background light cancelling method. Moreover, the QE of 55% at 940 nm is obtained. From this work, we successfully confirm the multi-tap lock-in pixel range sensor with high QE at NIR region.

## Figures and Tables

**Table 1:** Performance summary.

Parameter	Value
Process	0.2 µm SOI–CMOS technology
Pixel size	36 µm × 18µm
Fill factor	100% (Backside illumination)
Substrate thickness	200 µm
Modulation	Light and Gate Pulse Width: 40 ns Cycle time of light pulse: 520 ns Duty ratio: 7.7%
Modulation contrast	71% with 930 nm short-pulse laser
Light source	Wavelength: 940 nm Power density: 7.2 W/m^2^ @ 30 m
Integration time	520 μs (1000 cycle)
Quantum efficiency	55% (at 940 nm)
Linearity error	+1.8~−3.0%
Range resolution	4.5 cm (at 27 m, outdoor (75klux))
